# Cannabis-Induced Anxiety Disorder in the Emergency Department

**DOI:** 10.7759/cureus.38158

**Published:** 2023-04-26

**Authors:** Man Yee Keung, Erin Leach, Kaitlin Kreuser, Bradley W Emmerich, Steven Ilko, Matthew Singh, Thomas Sapp, Mariah Barnes, Lindsey Ouellette, Jeffrey S Jones

**Affiliations:** 1 Emergency Medicine, Michigan State University College of Human Medicine, Grand Rapids, USA; 2 Emergency Medicine, Spectrum Health Lakeland, Saint Joseph, USA; 3 Emergency Medicine, Spectrum Health - Michigan State University, Grand Rapids, USA; 4 Emergency Medicine, Spectrum Health Medical Group, Grand Rapids, USA

**Keywords:** panic disorders attack, clinical features, anxiety, toxicity, cannabis

## Abstract

Background: In December 2018, Michigan became the 10th state to legalize marijuana for adults. Since this law took effect, increased availability and use of cannabis in Michigan have led to increased emergency department (ED) visits associated with the drug's psychiatric effects.

Objectives: To describe cannabis-induced anxiety disorder's prevalence, clinical features, and disposition in a community-based study.

Methods: This was a retrospective cohort analysis of consecutive patients diagnosed with acute toxicity related to cannabis use (ICD-10 code F12). Patients were seen at seven EDs over a 24-month study period. Data collected included demographics, clinical features, and treatment outcomes in ED patients who met the criteria for cannabis-induced anxiety disorder. This group was compared to a cohort experiencing other forms of acute cannabis toxicity. Chi-squared and t-tests were used to compare these two groups across key demographic and outcome variables.

Results: During the study period, 1135 patients were evaluated for acute cannabis toxicity. A total of 196 patients (17.3%) had a chief complaint of anxiety, and 939 (82.7%) experienced other forms of acute cannabis toxicity, predominantly symptoms of intoxication or cannabis hyperemesis syndrome. Patients with anxiety symptoms had panic attacks (11.7%), aggression or manic behavior (9.2%), and hallucinations (6.1%). Compared to patients presenting with other forms of cannabis toxicity, those with anxiety were likelier to be younger, ingested edible cannabis, had psychiatric comorbidities, or had a history of polysubstance abuse.

Conclusions: Cannabis-induced anxiety occurred in 17.3% of ED patients in this community-based study. Clinicians must be adept in recognizing, evaluating, managing, and counseling these patients following cannabis exposure.

## Introduction

Cannabis is one of the most commonly used (and abused) recreational drugs in the United States, with approximately 18% of Americans, or 48.2 million people, reporting having used it in 2019 [1]. Michigan became the 10th state (and the first in the Midwest) to legalize cannabis for adults in December 2018. Increasing accessibility and use of cannabis in Michigan since the enactment of this law has led to increasing numbers of hospital visits that are related to the drug's adverse psychiatric effects [2]. Cannabis use for recreational purposes is frequently said to cause a euphoric feeling, increased sociability, and reduced anxiety. It can also cause adverse psychiatric effects such as panic disorder, anxiety, paranoia, and mania [3-5]. Our study sought to describe the clinical features, prevalence, and disposition of patients with cannabis-induced anxiety disorder in a community-based study utilizing seven emergency departments (EDs).

This article was previously presented as a meeting abstract at the American College of Emergency Physicians (ACEP) Research Forum, San Francisco, CA, in October 2022.

## Materials and methods

Study design/setting

We conducted a retrospective cohort analysis of all ED patients with cannabis-associated diagnostic codes (ICD-10 code F12) that presented to a hospital over a 24-month study period (November 2018-October 2020). Patients with acute cannabis-induced anxiety were compared to a larger sample of patients suffering from other types of acute cannabis toxicity. Affiliated institutions included three university-affiliated hospitals, three rural hospitals, and a pediatric acute care children's hospital spanning thirteen counties in West Michigan. The rural medical centers were not located within a metropolitan area as defined by the US Office and Management and Budget and the US Census Bureau. The combined annual ED census during the study period ranged from 268,000 to 273,000. An institutional review board (IRB) at each affiliated institution approved this retrospective observational study.

Inclusion and exclusion criteria

Adolescent and adult patients (age > 12 years) presenting to the ED with acute cannabis toxicity were identified using ICD-10 diagnostic codes. Our study cohort included patients fitting the DSM-5-TR (Diagnostic and Statistical Manual of Mental Disorders, Text Revision) criteria for cannabis-induced anxiety disorder [6]. These patients had a chief or presenting complaint of anxiety or panic attacks that occurred during or soon after cannabis intoxication. The anxiety caused clinically "significant distress or impairment in occupational, social, other important areas of functioning" [6]. Patients were excluded from the study cohort if they had evidence of psychosis or delirium or if "symptoms were better accounted for by an anxiety disorder that was not substance-induced" [6]. Our study cohort was then compared to the larger sample of ED patients with other forms of cannabis toxicity.

Study definitions

Cannabis toxicity refers to the negative effects of consuming excessive cannabis or when the drug adversely interacts with the body. Symptoms of cannabis toxicity can vary but may include anxiety, paranoia, hallucinations, impaired coordination, and decreased judgment [5]. In severe cases, it can cause severe nausea, vomiting, and even coma. The severity of symptoms depends on factors such as the amount of cannabis consumed, the potency of the drug, the individual's tolerance, and any underlying health conditions [5]. For this analysis, cannabis intoxication was defined as " perception alterations, mild motor impairment, euphoria, and intensification of ordinary sensory experiences" [6].

Data collection

All data were collected by medical students trained in study methodology and were blinded to the study objectives. The research staff was trained in data abstraction using a set of mock case records. One investigator oversaw data abstraction and confirmed that variable definitions were correctly applied. Research staff reviewed the free-text of ED records to determine if patient symptoms and clinical findings met pre-determined definitions of cannabis toxicity (e.g., panic attack, anxiety disorder, hyperemesis, psychosis). Abstracted data included demographics, presenting complaints, clinical findings, and treatment outcomes in cannabis patients with a chief complaint of anxiety. We then compared this group to a patient cohort suffering from other types of acute cannabis toxicity.

Statistical analyses

The primary outcome measures were the frequency and type of cannabis-induced anxiety symptoms documented in ED patients. Data were entered into a Microsoft Excel database (Microsoft Corp, Redmond, WA, USA). All analyses were performed using SAS statistical software (SAS Institute, Cary, NC, USA). One investigator performed a blinded critical review of a random sample of 10% of the medical records to determine the inter-rater reliability of data collection using the Kappa reliability test. Inter-rater reliability focused on the components used to adjudicate the DSM-5-TR criteria of cannabis-induced anxiety disorder. Descriptive statistics described means, standard deviation, and frequency distributions. T-tests and chi-squared tests were used to compare the main demographic and clinical course variables between our two patient groups: those with cannabis-induced anxiety and those with other types of acute cannabis toxicity. Duration of symptoms and ED length-of-stay data were compared with the Mann-Whitney U test. We chose a p-value <0.01 for statistical significance to decrease the risk of a type I error that may occur when many statistical tests are performed [7].

## Results

One thousand one hundred thirty-five children and adults were evaluated for acute cannabis toxicity during the study period. We found 196 patients (17.3%) had a chief complaint of severe anxiety or panic attacks that fit our inclusion criteria for cannabis-induced anxiety disorder. In comparison, 939 (82.7%) experienced some other form of acute cannabis toxicity, predominantly symptoms of intoxication or cannabis hyperemesis syndrome (Table 1).

**Table 1:** Patient Demographics and Outcomes

	Cannabis-induced Anxiety (N=196)	Other forms of Cannabis Toxicity (N=939)	P-value
Age (years), Mean (SD)	25.2 ± 9.6	28.5 ± 8.5	< 0.001
Gender (% female)	115 (58.7%)	461 (49.1%)	0.015
Race (% white)	124 (63.2%)	545 (58.0%)	0.179
Duration of symptoms (hours), median (IQR)	6.1 (2.3 to 12.5)	15.2 (3.6 to 16.9)	< 0.001
Edible cannabis	51 (26.0%)	106 (11.2%)	< 0.001
Medical comorbidities	34 (17.3%)	115 (12.2%)	0.054
Psych comorbidities	37 (18.9%)	96 (10.2%)	< 0.001
Polysubstance abuse	40 (20.4%)	123 (13.1%)	0.008
Physical restraints	12 (6.1%)	33 (3.5%)	0.089
Length of stay (hours), median (IQR)	2.3 (1.1 to 3.0)	3.0 (1.2 to 4.5)	0.063
Outcomes:			
Admitted	15 (7.7%)	76 (8.1%)	0.851
Psychiatric transfer	24 (12.2%)	34 (3.6%)	< 0.001
Jail	5 (2.6%)	9 (1.0%)	0.070

Intoxicated patients presented predominantly with lethargy, dizziness, confusion, and drowsiness. However, many also had secondary gastrointestinal (40.2%), respiratory (5.5%), neurologic (5.2%), and cardiovascular (3.9%) complaints.

Patients with cannabis-induced anxiety experienced panic attacks (13.3%), aggressive or manic behavior (9.2%), depression (4.6%), and suicidal ideation (3.1%). Most patients (64.8%) also had associated cardiopulmonary complaints, such as chest discomfort, dyspnea, tachycardia, and hypertension (Table 2).

**Table 2:** Symptoms associated with cannabis-induced anxiety disorder (N=196)

Neuropsychiatric Symptoms	
Severe anxiety	188 (98.9%)
Altered mental status	29 (14.8%)
Panic attack	26 (13.3%)
Aggression or manic behavior	18 (9.2%)
Tremulous, dizziness	18 (9.2%)
Paranoia	10 (5.1%)
Headache	10 (5.1%)
Depression	9 (4.6%)
Syncope	7 (3.6%)
Suicidal ideation	6 (3.1%)
Homicidal ideation	4 (2.0%)
Ataxia	4 (2.0%)
Speech difficulties	3 (1.5%)
Cardiopulmonary Symptoms	
Tachycardia	92 (46.9%)
Chest discomfort	37 (37.2%)
Dyspnea	35 (17.9%)
Hypertension	15 (7.7%)
Tachypnea	13 (6.6%)
Palpitations	11 (5.6%)
Cough	10 (5.1%)
Diaphoresis	7 (3.6%)
Wheezing	7 (3.6%)
Hypotension	2 (1.0%)

However, there were no ischemic events, unstable angina, or serious tachyarrhythmias. Patients presenting with anxiety, when compared to patients presenting with other forms of acute cannabis toxicity, were more likely to have consumed edible cannabis (26.0 vs. 11.2%, p <0.001), had psychiatric comorbidities (18.9 vs. 10.2%, p<0.001), were younger (25.2 vs. 28.5 years, p<0.001), and had a history of polysubstance abuse (20.4 vs. 13.1%, p=0.008). ED length of stay and hospital admission rates were at similar levels (8.7% vs 9.3%, p=0.79). Not surprisingly, more patients in the anxiety group were transferred to a psychiatric hospital (12.2% vs. 3.6%, p<0.001). The reliability of data collection (k = 0.86) showed excellent agreement.

## Discussion

This study conducted a retrospective analysis of seven Emergency Departments in West Michigan and found that 17.3% of patients experienced cannabis-induced anxiety disorder after acute exposure to cannabis. These results align with a prior study of clinical presentations on acute cannabis toxicity from Switzerland and a study from the European Drug Emergencies Network, which used data from 36 centers in 24 European countries [8,9]. Following cannabis legalization in Colorado, a statewide database demonstrated a "fivefold higher prevalence of mental health diagnoses in cannabis-associated ED visits" from 2012 to 2014 [10]. Overall, 31% of ED visits relating to acute cannabis toxicity had mental health primary diagnoses [10].

A recent meta-analysis of ten prospective studies found that cannabis users are likelier to develop an anxiety disorder, with a pooled odds ratio of 1.25 and a confidence interval of 95% (1.01 to 1.54) [4]. The same review also examined 14 additional longitudinal studies that might otherwise be excluded from systematic reviews. While these longitudinal studies reported mixed findings, many indicated a positive association between cannabis use and an increased risk of developing an anxiety disorder or increased severity of the condition [4]. These results reinforce previous meta-analyses, which despite significant methodological flaws, reported pooled ORs between 1.15 and 1.28, also indicating a positive association between cannabis use and anxiety disorders [11-13]. Finally, one prospective study demonstrated that a reduction in cannabis use among individuals treated for cannabis use disorder was associated with an improvement in the severity of anxiety [14].

Despite the public perception of the safety of cannabis, an increasing number of anxiogenic disorders have been documented in temporal relation to cannabis use [2-5, 8, 9]. These have included panic attacks, paranoia, depression, suicidal ideation, behavioral crises, and even homicidal ideation (Table 2). The psychoactive constituents in marijuana, of which D9-tetrahydrocannabinol (THC) is the main ingredient, disrupt the innate functioning of the endocannabinoid system, which plays a vital role in anxiety, depression, cognition, and many other functions [15-17]. The word cannabinoid refers to any chemical substance, regardless of origin or structure, that binds to the cannabinoid receptors of the body and brain [17]. The pathophysiology by which this plant causes an anxiogenic effect in some individuals is poorly understood. Still, given that cannabis contains over 400 different chemical compounds, including 100 different cannabinoids, the pathology is likely to involve multiple pathways [18-19]. Teenagers and young children, whose brains are still developing, are particularly vulnerable to marijuana's neuropsychiatric effects [8-9]. A recent review by Petrie et al. discusses the evidence describing how endocannabinoids and exogenous cannabinoids regulate the initiation and termination of anxiety states [19]. Lastly, the study of pharmacogenomics has become a valuable aspect of ongoing cannabis research [3]. Variability in cannabinoid transporter genes, receptor genes, pharmacokinetics (drug absorption, distribution, metabolism, and elimination), and pharmacodynamics are essential for future cannabis research.

The neuropsychiatric toxicity of cannabis is also affected by age, comorbidities, dose, and type of cannabinoids ingested, duration of use, as well as associated use of other illicit substances [2-4, 15]. In addition, given the expanding use of cannabis edibles and the likelihood that they have considerable levels of D9-tetrahydrocannabinol (THC), clinicians should also inquire about food or beverage products infused with cannabis extract [5]. Synthetic cannabinoids, such as K2 and Spice, are also gaining popularity. These designer drug molecules are typically sprayed onto plant matter and then smoked. These compounds have greater affinity and efficacy at cannabinoid CB1 receptors and therefore are more potent than natural cannabis and may have correspondingly more psychoactive severe effects [20]. It has been reported that by altering the chemical compound of THC, more than 700 synthetic compounds have been produced with greater binding affinity and potency to cannabinoid receptors [20]. Many of these synthetic compounds have active metabolites that significantly increase the risk of anxiety symptoms and disease. Confounding the problem of cannabis neurotoxicity, legally sold marijuana may be contaminated with pesticides, heavy metals, and foreign matter. These contaminants may affect the mechanism of action and pharmacokinetics of cannabinoids and, thus, potentially alter their clinical features [21].

There were several limitations in our study. It is difficult to establish causality in observational studies because factors other than cannabis use may be directly associated with the risk of anxiety and panic attacks. In addition, confounding factors could predispose a person to cannabis use and psychiatric illness. This makes it difficult to confidently attribute the increased risk of anxiety to cannabis use [22]. As with any review of medical records, there is always inconsistency in the assessment and documentation by different clinicians. Thus, the description of outcome variables may not have been consistent. Drug use reporting varied substantially among our patients and was highly subjective (response bias). Drug screening was not performed in all patients; it was not felt prudent by clinicians if patients were readily admitted to drug use and symptoms were consistent with cannabis toxicity.

Considerable controversy still exists about how this anxiety disorder should be defined, limiting external validity and making comparisons between studies challenging. Our sample population was drawn from thirteen counties located in the Midwestern United States. Although we utilized a mix of rural and urban hospitals for our study population, it's unclear if our sample accurately represents patients from other regions. Despite this limitation, our study provides valuable insights into the risks of using cannabis for recreational purposes, building on previous research. Finally, cannabis-related toxicity accounts for a small but growing percentage of emergency medical services (EMS) that provide urgent pre-hospital treatment. Depending on the co-ingestion of alcohol or other drugs, up to 37% of patients may be managed by paramedics on the scene and not transported to the hospital [23]. Therefore several patients with cannabis-induced anxiety may not have presented to our ED.

## Conclusions

In this retrospective analysis in seven EDs in West Michigan, cannabis-induced anxiety was common after acute or chronic cannabis exposures, occurring in 17.3% of patients. Because of the growing utilization of cannabis in our society, healthcare providers must educate and involve patients in a risk/benefit discussion concerning its use. Cannabis users, especially those with psychiatric comorbidities, should be counseled appropriately when presenting with psychiatric symptoms. ED clinicians may use these findings to educate patients about the dangers of continued cannabis use and should consider cannabis use as an aggravating factor for anxiety and panic attacks. Concomitant drug and alcohol use should also be reviewed since these patients are more likely to have a history of polysubstance abuse. Patients with acute panic attacks, new onset anxiety, or unexplained behavioral changes should have a detailed drug history. If a patient is not honest about their drug use, obtaining a urine drug test may be helpful. Lastly, public health policy-makers and legislatures must be informed of the growing evidence regarding the adverse outcomes of cannabis use on mental health. Research has been restricted, given the controversial legal history surrounding cannabis. Evidence-based research and prospective studies on the mental-health effects of cannabis use will be needed as more state legislatures approve the legalization of marijuana.
